# Economic evaluation studies in the field of HIV/AIDS: bibliometric analysis on research development and scopes (GAP_RESEARCH_)

**DOI:** 10.1186/s12913-019-4613-0

**Published:** 2019-11-14

**Authors:** Bach Xuan Tran, Long Hoang Nguyen, Hugo C. Turner, Son Nghiem, Giang Thu Vu, Cuong Tat Nguyen, Carl A. Latkin, Cyrus S. H. Ho, Roger C. M. Ho

**Affiliations:** 1 Department of Health Economics, Institute for Preventive Medicine and Public Health, Hanoi Medical University, Hanoi, Vietnam; 2 Bloomberg School of Public Health, Johns Hopkins University, Baltimore, MD USA; 3 Center of Excellence in Behavioral Medicine, Nguyen Tat Thanh University, Ho Chi Minh City, 70000 Vietnam; 4 Oxford University Clinical Research Unit, Ho Chi Minh City, 70000 Vietnam; 5 Centre for Applied Health Economics, Griffith University, Brisbane, Australia; 6 Center of Excellence in Evidence-based Medicine, Nguyen Tat Thanh University, Ho Chi Minh City, 70000 Vietnam; 7 Institute for Global Health Innovations, Duy Tan University, Da Nang, Vietnam; 8 Department of Psychological Medicine, National University Hospital, Singapore, Singapore; 9 Department of Psychological Medicine, Yong Loo Lin School of Medicine, National University of Singapore, Singapore, 119228 Singapore; 10 Biomedical Global Institute of Healthcare Research & Technology (BIGHEART), National University of Singapore, Singapore, 117599 Singapore

**Keywords:** HIV/AIDS, Health economics, Economic evaluation, Bibliometric, Content analysis

## Abstract

**Background:**

The rapid decrease in international funding for HIV/AIDS has been challenging for many nations to effectively mobilize and allocate their limited resources for HIV/AIDS programs. Economic evaluations can help inform decisions and strategic planning. This study aims to examine the trends and patterns in economic evaluation studies in the field of HIV/AIDS and determine their research landscapes.

**Methods:**

Using the Web of Science databases, we synthesized the number of papers and citations on HIV/AIDS and economic evaluation from 1990 to 2017. Collaborations between authors and countries, networks of keywords and research topics were visualized using frequency of co-occurrence and Jaccards’ similarity index. A Latent Dirichlet Allocation (LDA) analysis to categorize papers into different topics/themes.

**Results:**

A total of 372 economic evaluation papers were selected, including 351 cost-effectiveness analyses (CEA), 11 cost-utility analyses (CUA), 12 cost-benefit analyses (CBA). The growth of publications, their citations and usages have increased remarkably over the years. Major research topics in economic evaluation studies consisted of antiretroviral therapy (ART) initiation and treatment; drug use prevention interventions and prevention of mother-to-child transmission interventions. Moreover, lack of contextualized evidence was found in specific settings with high burden HIV epidemics, as well as emerging most-at-risk populations such as trans-genders or migrants.

**Conclusion:**

This study highlights the knowledge and geographical discrepancies in HIV/AIDS economic evaluation literature. Future research directions are also informed for advancing economic evaluation in HIV/AIDS research.

## Background

Global efforts to put an end of the HIV/AIDS epidemic in 2030 require extraordinary amounts of investments in both international and national levels [1]. The latest global statistics in 2019 reported that in 2018, more than 37.9 million people are currently living with HIV/AIDS, and 770 thousand people died due to AIDS-related diseases [2]. African countries continue to have the highest number of people living with HIV (PLWH) with 27.8 million, following Asian and the Pacific region with 5.9 million [2]. It has been estimated that from 2000 to 2015, worldwide expenditures on HIV/AIDS totaled US$ 562.6 billion, of which national expenditure accounted for 57.6% [1]. The Joint United Nations Programme on HIV/AIDS (UNAIDS) estimates that by 2020, total resources needed for HIV/AIDS responses in low and middle-income countries (LMICs) will be a sum of US$ 26.2 billion, which is US$ 4.9 billion higher than the investment in 2017 (US$ 21.3 billion) [3]. Filling this financial resource gap in these countries becomes a significant challenge as they are shifting to self-sustain financing HIV/AIDS programs due to a rapid decrease of foreign aids [3–5]. With limited available resources, selecting optimal allocation strategies are vital to achieving the highest benefits with the lowest costs, or in other words, focus on the right population, in the right place, and at the right time [6–8].

Economic evaluations can support this decision-making process by systematically quantifying and comparing the costs and outcomes of different interventions or health programs. Economic evaluation has been defined as “the comparative analysis of alternative courses of action in terms of both their costs and consequences” [9]. There are three primary forms of economic evaluation: cost-effectiveness analysis (CEA), cost-benefit analysis (CBA) and cost-utility analysis (CUA) [9]. They are distinguished by the measure of outcomes. The first form measures natural units of effects (e.g., new HIV infection averted or prevented), while the second form evaluates the outcomes in monetary terms, and the third form assesses the effectiveness in terms of Disability-adjusted life years (DALYs) or Quality-adjusted life years (QALYs) [9–11]. In the literature, economic evaluation is proposed as a powerful tool to assist in prioritization and scarce resources allocation [8, 12, 13]. In the field of HIV/AIDS, the number of economic evaluation studies significantly increase in recent years with a wide range of topics from prevention (e.g., behavior risk reduction, HIV testing and screening, pre-prophylaxis exposure, or prevention of mother-to-child HIV transmission) [8, 14–18] to treatment and care (e.g., ART alone, ART with other medications, or adherence support) [19–22]. The rapid development of economic evaluation studies in this field requires a further assessment to understand the knowledge gap and propose future research directions.

Importantly, one question that should be raised is about the applicability and transferability of the economic evaluation studies from one setting to others, considering both technical and contextualization aspects. Ramos et al. indicated that scientific production on HIV was dominated by the United States of America (USA) and Western Europe (accounted for 83% of total publications in 2003), while little empirical evidence was available in the most severe HIV-affected regions such as Sub-Sahara Africa or South East Asia [23]. This finding was also confirmed by other narrative and systematic reviews on economic evaluation in HIV/AIDS [7, 8, 17, 24–26]. A prior analysis in some low- and middle-income countries (LMICs) found that human capacity was a major limiting attributed to the unmet need of health economic evidence for decision-making process [27]. Indeed, countries with lower human capacity may be beneficial if they can adapt and apply the economic evaluation evidence created from neighboring nations in the same region, or with the similar socioeconomic, health system, and decision-making processes [28, 29]. Thus, examining collaboration networks among countries is helpful on the transferability of economic evaluation evidence and the enhancement of the countries’ capacities to build economic evaluation programs in the field of HIV/AIDS.

Bibliometric analysis is a useful tool to explore the research gaps and collaboration networks in a particular research field [30, 31]. This method informs objective data about scientific publications in both quantity and quality perspectives [32]. Bibliometric studies show the development of scientific publications over time, including the number of publications; the emergence of new scientific terms and journals; topics, subject areas and contents of research; and the impact of research via citations and co-citations. Moreover, this approach enables scholars to understand the collaboration patterns by identifying the geographic distribution and co-authorships among institutions and countries [30, 31]. Analyzing these characteristics would help to figure out the current state and further agenda for economic evaluation in HIV/AIDS research, which can be eventually used to advance these research areas.

Previously, several papers attempted to provide the health economic research patterns in HIV/AIDS. For example, Youle et al. searched five databased and extracted 79 economic studies (including cost analysis and economic evaluations) published from 1985 to 1998 [33]. Other two studies were conducted by Beck et al. to review publications about cost of HIV/AIDS treatment and care [34, 35]. However, to date, little is known about the patterns of research development and scopes in scientific publications, as well as collaborations in economic evaluation studies of HIV/AIDS interventions and programs. This study aims to examine the trends and patterns in economic evaluation studies in the field of HIV/AIDS and determine their research landscapes.

## Methods

### Search strategy

The Web of Science (WoS) was employed to collect research articles regarding economic evaluations in HIV/AIDS. We preferred WoS instead of other databases namely Scopus or MEDLINE due to its useful in examining research disciplines, which could not be performed in other databases. In addition, the WoS database encompassed high quality scientific journals, while other databases consisted of journals with varied quality [23–25]. The WoS also allowed us to conduct advanced search as well as filter the result according to specific criteria in order to assess the research productivity in different subgroups. In this study, we concentrated on publications regarding economic evaluations in HIV/AIDS published until December 31st, 2017 in the WoS journals. We did not include 1) non-English literature; 2) grey literature, conference proceedings, or books/book chapters. We also excluded narrative reviews or systematic reviews or meta-analysis. The search strategy was performed in three steps:

- First, we combined the following terms to extract publications about HIV/AIDS using option “Topic”: HIV; human-immunodeficiency-virus; AIDS; Acquired-Immune-Deficiency-Syndrome
- Second, used search term: “cost-effectiveness”, “cost effectiveness”, “cost-benefit”, “cost benefit”, “cost utility” or “cost-utility” in the Title or Abstract to extract publications about economic evaluation.
- Final, we screened the titles and abstracts of these publications according to eligible and exclusion criteria in order to extract only those meeting our inclusion criteria.

### Data extraction

**Fig. 1:**
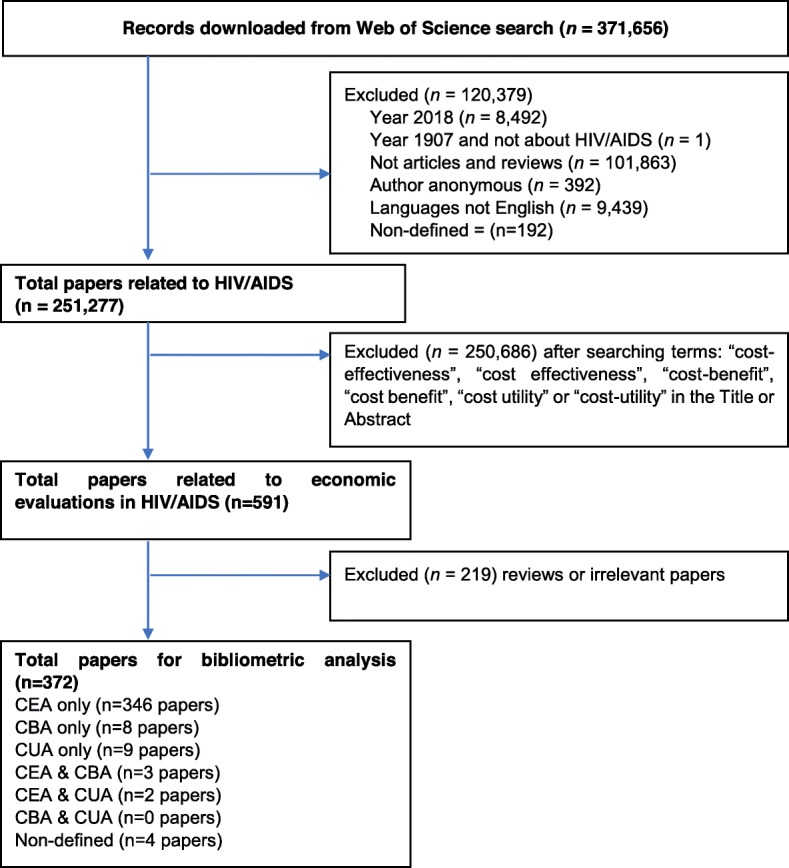
Selection of papers

Publication data were downloaded from the WoS comprising all necessary information about authors’ names, title of papers, journal names, keywords, institutional affiliations, the frequency of citation, subject category, usage (number of downloads) and abstracts. All data were collected and synthesized using Microsoft Excel to check for errors before further analysis. Standardized procedures were developed and performed by two researchers to check for any discrepancies in author names and affiliations, then make necessary changes to make the search results consistent. Disagreements between the two researchers were resolved by discussing with a senior researcher. After having correct data, we screened the titles and abstracts, and excluded publications which were: 1) not original articles and reviews; 2) not published in English; 3) not about HIV/AIDS. Figure 1 shows the results of the searching process. The final dataset was transferred to a data file to be further analyzed using STATA version 14.0 (STATACorp., Texas, the USA).

### Data analysis

We described some fundamental characteristics of publications, which consisted of years of publication, the number of papers per country/per year, total citations up to 2017, mean citation rate per year, total usage in the last 6 months/5 years, and mean use rate the last 6 months/5 years. We used Circos platform to draw network graphs to figure the collaboration networks among countries using co-authorships data [27]. This tool was originally used to figure the variations of genome [27]. We used the VOSviewer (version 1.6.8, Center for Science and Technology, Leiden University, the Netherlands) to illustrated networks of co-occurrence keywords. By using STATA version 14, we conducted a Latent Dirichlet Allocation (LDA) analysis to categorize papers into different topics/themes [36–40]. Two researchers reviewed all titles and abstracts of papers in each topic/theme and identify the content of the ten topics. Disagreements were resolved by involving opinions from a senior researcher. Jaccard’s similarity index was used to determine research topics or terms that most frequently co-occur with each other.

## Results

Figure 1 reveals the searching and selection processes. Among 250,270 papers about HIV/AIDS, 372 papers are about economic evaluations in HIV/AIDS.

Table 1 outlines some basic characteristics of the selected publications. Overall, the number of articles studying economic or finance perspective in the field of HIV/AIDS, as well as the total citations and mean cite rate per year increased significantly from period 1991–1997 to 1998–2001 albeit a decrease in the period 2002–2005. Notably, the total usage (total number of downloads) and the mean use rate in the last 5 years of papers published in 2013 were the highest compared with other years.

**Table 1:** General characteristics of publications

Year published	Total number of papers	Total citations	Mean cite rate per year	Total usage last 6 month^a^	Total usage last 5 years^a^	Mean use rate last 6 month^b^	Mean use rate per year in last 5 year^b^
2017	23	25	1.09	36	82	1.57	0.71
2016	26	99	1.90	13	138	0.50	1.06
2015	23	144	2.09	9	143	0.39	1.24
2014	25	224	2.24	8	170	0.32	1.36
2013	28	369	2.64	10	285	0.36	2.04
2012	26	494	3.17	3	211	0.12	1.62
2011	26	396	2.18	6	177	0.23	1.36
2010	16	498	3.89	1	105	0.06	1.31
2009	10	119	1.32	1	23	0.10	0.46
2008	14	487	3.48	1	66	0.07	0.94
2007	11	171	1.41	0	34	0.00	0.62
2006	21	527	2.09	10	107	0.48	1.02
2005	10	765	5.88	2	43	0.20	0.86
2004	13	362	1.99	4	45	0.31	0.69
2003	8	438	3.65	3	32	0.38	0.80
2002	3	60	1.25	1	16	0.33	1.07
2001	17	1023	3.54	1	68	0.06	0.80
2000	17	880	2.88	2	67	0.12	0.79
1999	15	626	2.20	1	39	0.07	0.52
1998	14	519	1.85	1	24	0.07	0.34
1997	7	507	3.45	2	40	0.29	1.14
1996	7	250	1.62	0	10	0.00	0.29
1995	3	69	1.00	0	2	0.00	0.13
1994	5	87	0.73	2	4	0.40	0.16
1993	2	27	0.54	0	0	0.00	0.00
1992	1	10	0.38	0	0	0.00	0.00
1991	1	109	4.04	0	0	0.00	0.00

^a^Total usage: Total number of downloads

^b^Use rate: Total number of downloads/Total number of papers

**Fig. 2:**
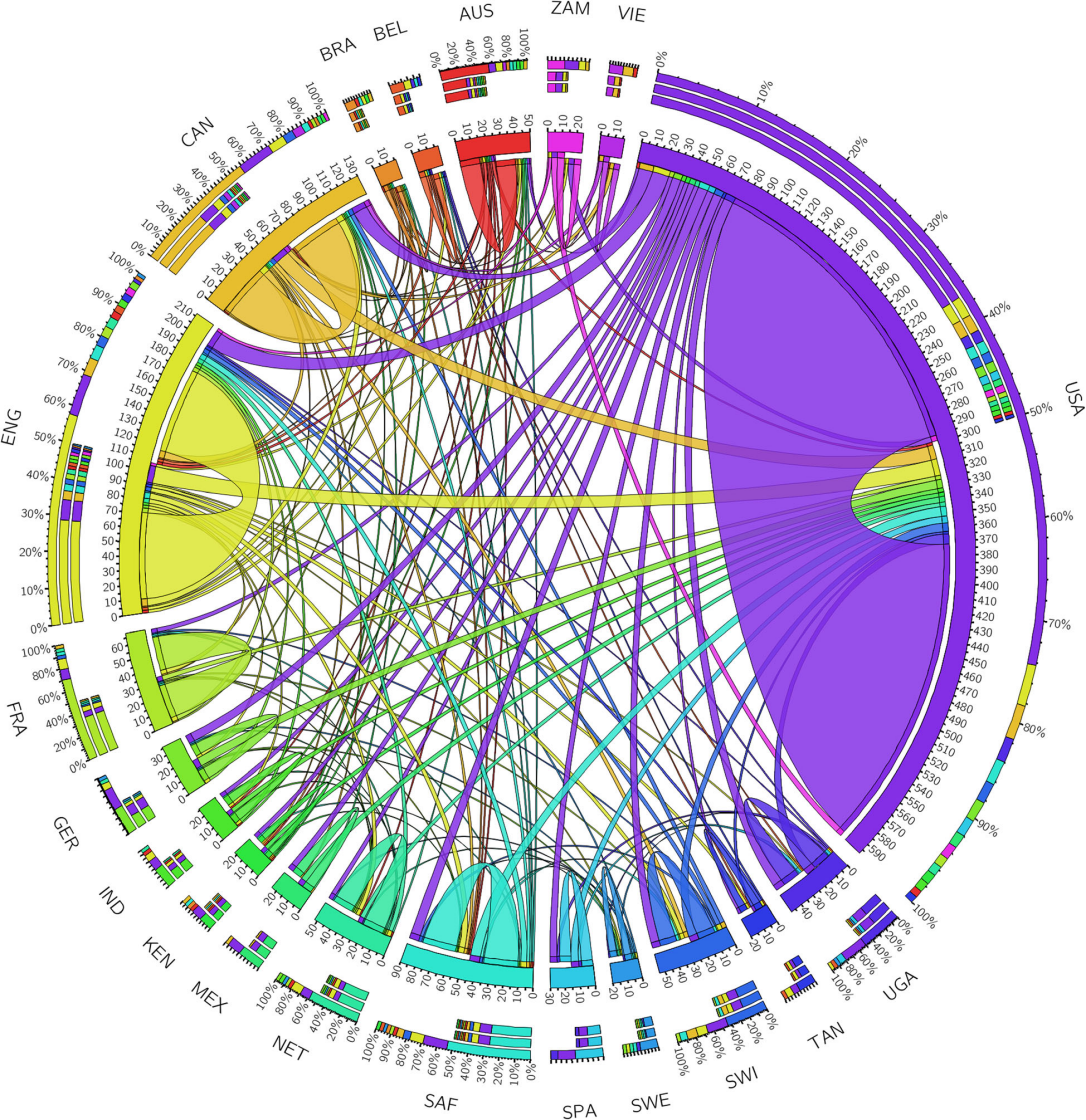
Collaboration network between the top 20 countries by the number of publications. The outer rim reflects the volume of collaborations between one country with other countries in the top 20, showing collaboration among countries. Abbreviation: USA, the United States of America; ENG, England; GER, Germany; AUS, Australia; NET, Netherlands; CAN, Canada; SAF, South Africa; FRA, France; SWI, Switzerland; UGA, Uganda; SPA, Spain; MEX, Mexico; IND, India; ZAM, Zambia, KEN, Kenya, TAN, Tanzania; BEL, Belgium; SWE, Sweden; BRA, Brazil; VIE, Vietnam

Figure 2 reveals the collaboration networks between the top 20 contributing countries. Overall, 46 countries contributed to at least one article in the final dataset with 372 papers, of which the United States of America (USA) and England had the highest number of publications (306 and 118 papers, respectively), following by Canada (66 papers), South Africa (45 papers), and France (35 papers). In top 10 countries with the highest number of publications, only South Africa and Uganda had hyperendemic and generalized HIV/AIDS epidemic, respectively, while others had concentrated (such as the USA or England) or low-level epidemic (such as France). Meanwhile, among the top 20 country, only South Africa, Uganda, Zambia, Kenya and Tanzania had hyperendemic or generalized epidemic. England and the USA had the highest volume of collaborations with 27 and 22 countries, respectively. These were followed by Canada (13 countries), South Africa (11 countries) and Australia (11 countries).

**Fig. 3:**
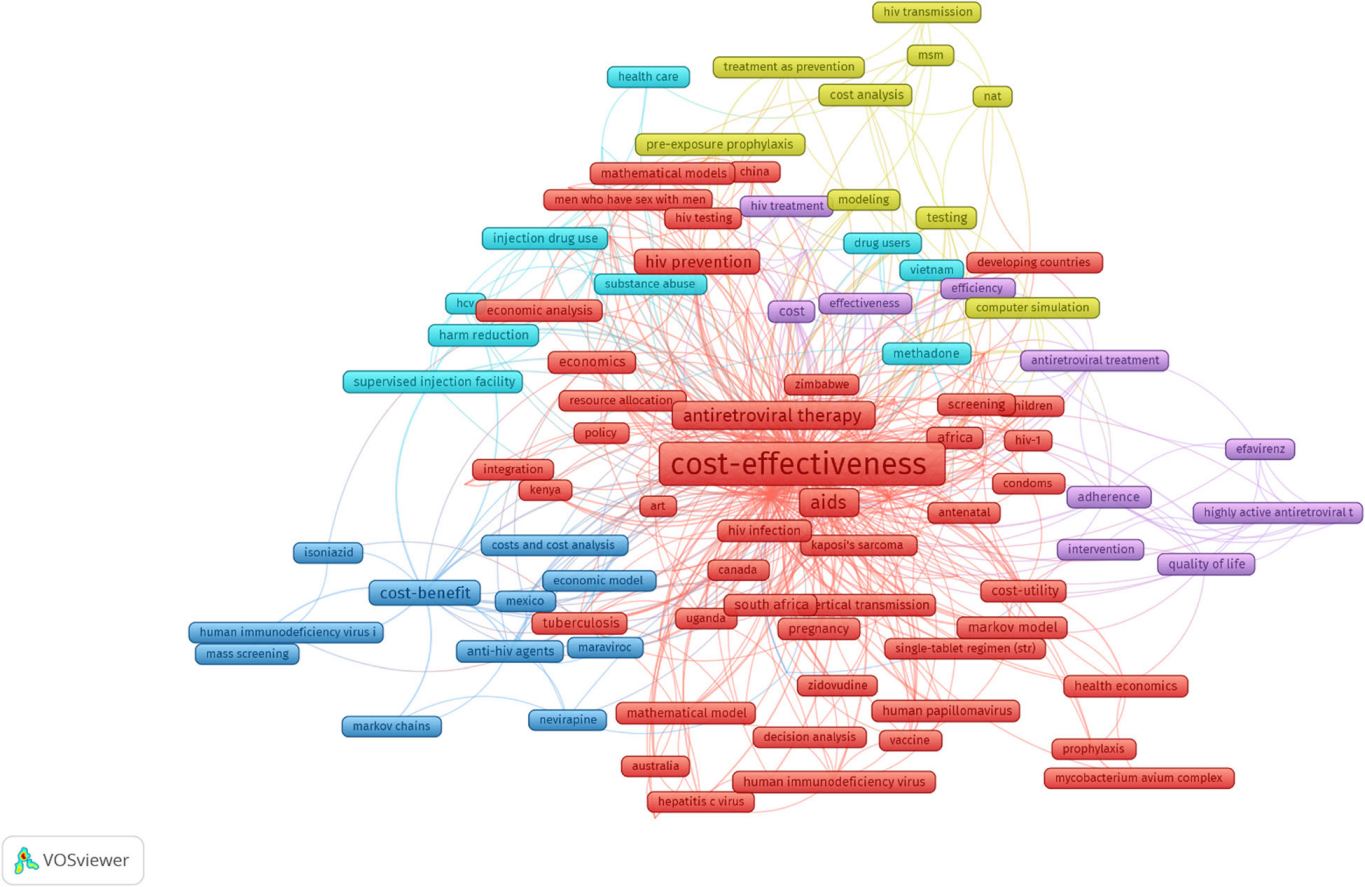
Co-occurrence of most frequent author’s keywords. Note: the colors refer to different clusters; the nodes size reflects keywords’ occurrences; lines’ thickness was visualized according to the strength of the relationship between two keywords

To illustrate the scopes of studies and development of research landscapes, we performed abstracts and keywords analysis. Figure 3 reveals five major clusters from 121 most frequent keywords co-occurrence. Each keyword had to appear at least 2 times. Of which, there were five greatest clusters of keywords: 1) the red cluster indicated topics of cost-effectiveness studies such as HIV and Sexually Transmitted Infections (STIs) prevention or HIV treatment in developing countries; 2) the yellow cluster indicated interventions using ART as prevention (such as pre-exposure prophylaxis or treatment as prevention approach), especially in men who have sex with men; 3) the blue cluster referred to the economic evaluation studies on injection drug use; 4) the navy blue cluster referred to topics of cost-benefit studies, focusing on HIV screening and treatment; 5) the purple cluster covered the interventions to improve adherence and quality of life in HIV patients.

Table 2 lists the top 10 research topics as well as the 20 most frequent keywords in each topic that emerged from the LDA for contents of titles/abstracts. The top three topics with highest number of publications were 1) Economic evaluations for ART interventions; 2) Economic evaluations for Drug use prevention interventions (such as harm reduction or methadone maintenance treatment); and 3) Economic evaluation for Prevention of mother-to-child transmission interventions.

**Table 2:** Ten research topics emerged classified by LDA for titles and abstracts’ contents

No	Topic	Most frequent terms	N	Percent
1	ART intervention	cost-effectiveness; antiretroviral; therapy; patients; analysis, clinical; effectiveness; treatment; objective; infection, highly; regimens; study; HIV-infected; combination, trial; active; HAART; HIV-1; adults	45	13.2%
2	Drug use prevention intervention	cost-effectiveness; treatment; methadone; maintenance; injection, users; health; programs; study; intervention, supervised; drugs; prevention; reduction; background, opioid; analysis; substance; abuse; injecting	41	12.0%
3	Prevention of mother-to-child transmission intervention	cost-effectiveness; transmission; Africa; objective; model; south; women; prevent; methods; effectiveness; sub-Saharan; mother-to-child; prevention; strategies; analysis; design; reduce; health; interventions; estimate	37	10.8%
4	Cancer screening and testing intervention in HIV/AIDS patients	Human; virus; immunodeficiency; cost-effectiveness; screening; Hepatitis; testing; blood; infection; study; compared; antibody; cancer; background; tests; cervical; donations; purpose; papillomavirus; determine	36	10.5%
5	ART intervention in resource-limited settings	antiretroviral; therapy; cost-effectiveness; settings; background, treatment; monitoring; health; Africa; resource-limited, clinical; sub-Saharan; guidelines; viral; resistance, world; different; first-line; organization; countries	35	10.2%
6	HIV counseling and testing intervention	testing; screening; cost-effectiveness; united; states, analysis; objective; voluntary; counseling; design, effectiveness; disease; routine; impact; evaluate, infection; patients; expanded; benefits; objectives	33	9.6%
7	Condom distribution intervention	cost-effectiveness; analysis; transmitted; workers; sexually, infections; female; background; south; condom, program; impact; Africa; cost-benefit; cost-utility, India; health; evidence; diseases; intervention	32	9.4%
8	HIV-related respiratory diseases treatment and prevention	Effectiveness; tuberculosis; background; HIV-infected; patients; strategies; infection; efficacy; diagnosis; mortality; Individuals; rapid; diagnostic; study; persons; early; preventive; infected; isoniazid; Uganda	28	8.2%
9	Pre and post-exposure prophylaxis interventions	cost-effectiveness; prophylaxis; economic; impact; evaluation, prevention; sexual; background; pre-exposure; intervention, program; potential; objective; post-exposure; exposure, Zambia; couples; following; service; methods	28	8.2%
10	HIV/AIDS vaccination intervention	costs; prevention; HIV/aids; cost-effectiveness; effectiveness, interventions; disease; economic; treatment; estimates, study; comparing; important; medical; policy; using; healthcare; integrated; incremental; vaccine	27	7.9%

**Fig. 4:**
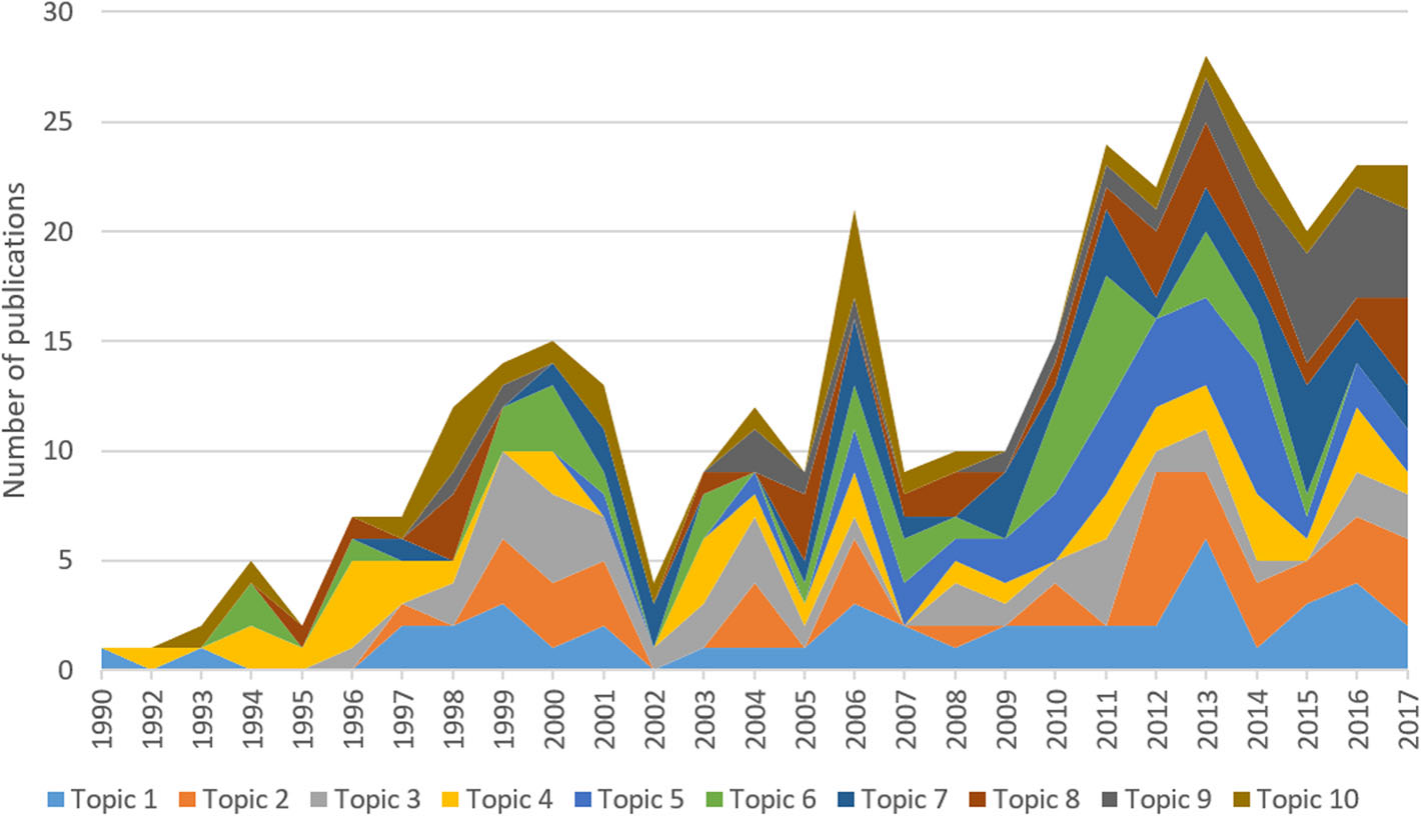
Changes in research topics development

Figure 4 showed the trend of research topics in the whole period. The first paper published in 1990 is belonged to Topic 1, following by a publication in 1992 about Topic 3. Since then, Topic 1 and Topic 2 were still paid the highest attention. However, in the last 3 years, publications in the Topic 9 “Pre and post-exposure prophylaxis interventions”, following by Topic 1, Topic 2 and Topic 5, have been raised significantly compared to other topics.

**Fig. 5:**
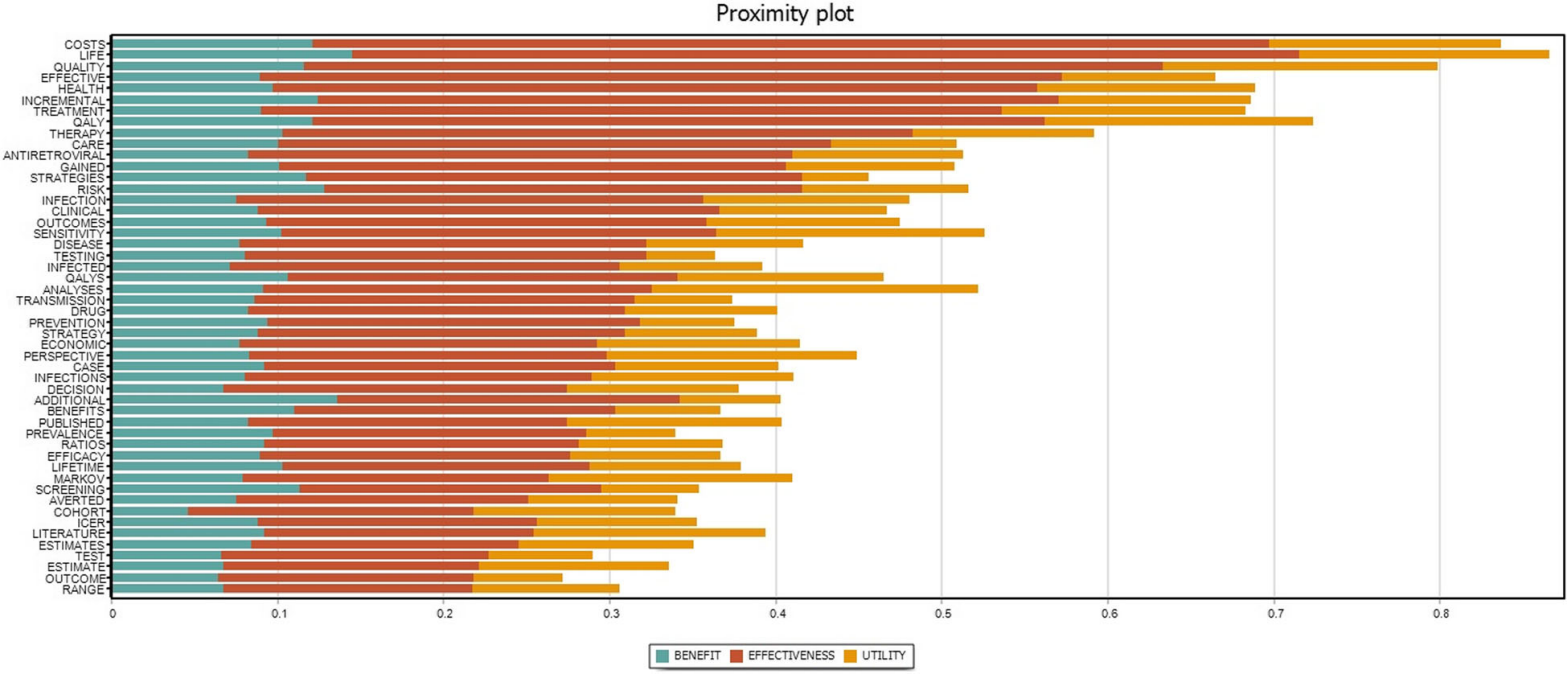
Proximity Plot of “cost-effectiveness analysis” (CEA), “cost-utility analysis” (CUA), “cost-benefit analysis” (CBA) terms with top 50 most frequent concurrence terms in 372 Economic Evaluation Studies’ abstracts. The x-axis shows the Jaccard coefficient which reflect similarities amning certain sample sets. It refers to the range of the intersection divided by the range of the union of the sample sets

Figure 5 describes the most frequent terms co-occurring with “cost-effectiveness analysis” (CEA), “cost-utility analysis” (CUA), “cost-benefit analysis” (CBA) terms in the content analysis of all abstracts. CBA has been identified that frequently co-occurred with strategies for risk reduction and screening and testing services. This method was more likely to be treated as an additional method along with CEA as the terms “life,” “quality,” “QALYs” were frequently presented. The Markov model seems to be a more common method in CBA compared to the Decision Tree. Meanwhile, in terms of CEA, HIV treatment and care were perhaps the most common topic, following by risk reduction strategies (especially drug use), and HIV screening and testing. QALY gained was the most common outcome, followed by infectious cases averted. Decision Tree and Markov Model were the two most common methods with similar shares in total publications.

Similar to CEA, treatment was the most common topic in CUA, following by risk reduction and care service. The Markov model was more frequently co-occurring with CUA compared to the Decision tree. Published papers and cohort studies were more likely to be the most common sources of data for all three forms of economic evaluation.

Top 20 most cited papers on economic evaluation in HIV populations are presented in Table 3. These studies primarily focused on medications for HIV treatment and care, behavior risk reduction (drug use), and HIV/Cancer screening and testing. Moreover, some studies concentrated on evaluating interventions to address co-infections such as Hepatitis C, Tuberculosis and other sexually transmitted infections and opportunistic infections. Other studies focused on different sub-populations such as breastfeeding women and men who have sex with men.

**Table 3:** Top 20 most cited papers among economic evaluation publications

#	Title	Journal	Citations	Years	Cite rate per year
1	Expanded screening for HIV in the United States - An analysis of cost-effectiveness [41]	New England Journal Of Medicine	369	2005	28.38
2	The cost effectiveness of combination antiretroviral therapy for HIV disease [42]	New England Journal Of Medicine	349	2001	20.53
3	Cost-effectiveness of voluntary HIV-1 counselling and testing in reducing sexual transmission of HIV-1 in Kenya and Tanzania [43]	Lancet	250	2000	13.89
4	Cost effectiveness of single-dose nevirapine regimen for mothers and babies to decrease vertical HIV-1 transmission in sub-Saharan Africa [44]	Lancet	196	1999	10.32
5	The cost-effectiveness of NAT for HIV, HCV, and HBV in whole-blood donations [45]	Transfusion	184	2003	12.27
6	Use of genotypic resistance testing to guide HIV therapy: Clinical impact and cost-effectiveness [46]	Annals Of Internal Medicine	169	2001	9.94
7	Updates of cost of illness and quality of life estimates for use in economic evaluations of HIV prevention programs [47]	Journal Of Acquired Immune Deficiency Syndromes And Human Retrovirology	167	1997	7.95
8	The cost-effectiveness of preventing AIDS-related opportunistic infections [48]	JAMA-Journal Of The American Medical Association	163	1998	8.15
9	HIV transmission and the cost-effectiveness of methadone maintenance [49]	American Journal Of Public Health	134	2000	7.44
10	Cost-Effectiveness of Serum Cryptococcal Antigen Screening to Prevent Deaths among HIV-Infected Persons with a CD4(+) Cell Count <= 100 Cells/mL Who Start HIV Therapy in Resource-Limited Settings [50]	Clinical Infectious Diseases	130	2010	16.25
11	Should resistance testing be performed for treatment-naive HIV-infected patients? A cost-effectiveness analysis [51]	Clinical Infectious Diseases	125	2005	9.62
12	Cost-effectiveness of improved treatment services for sexually transmitted diseases in preventing HIV-1 infection in Mwanza Region, Tanzania [52]	Lancet	124	1997	5.90
13	Cost-effectiveness of screening for anal squamous intraepithelial lesions and anal cancer in human immunodeficiency virus-negative homosexual and bisexual men	American Journal Of Medicine	121	2000	6.72
14	Positive and negative life events after counselling and testing: the Voluntary HIV-1 Counselling and Testing Efficacy Study [53]	AIDS	112	2001	6.59
15	Modeling the impact of HIV chemoprophylaxis strategies among men who have sex with men in the United States: HIV infections prevented and cost-effectiveness [54]	AIDS	110	2008	11.00
16	Cost-Effectiveness Of Low-Dose Zidovudine Therapy For Asymptomatic Patients With Human-Immunodeficiency-Virus (HIV) Infection [55]	Annals Of Internal Medicine	109	1991	4.04
17	Cost-effectiveness of expanded human immunodeficiency virus-testing protocols for donated blood [56]	Transfusion	105	1997	5.00
18	The cost-effectiveness of buprenorphine maintenance therapy for opiate addiction in the United States [57]	Addiction	98	2001	5.76
19	The cost-effectiveness of HLA-B*5701 genetic screening to guide initial antiretroviral therapy for HIV [58]	AIDS	89	2008	8.90
20	Is antenatal syphilis screening still cost effective in sub-Saharan Africa [59]	Sexually Transmitted Infections	85	2003	5.67

## Discussion

This study mapped the trends and patterns of economic evaluation publications in the field of HIV/AIDS, and hence it enriches the previous bibliometric studies regarding health economics in HIV/AIDS research [33–35]. Our study captured the growth of these publications as well as their usage in the last 5 years, suggesting the critical role of this topic in HIV/AIDS research. We also observed the overwhelming majority of CEA compared to CUA and CBA; and the predominance of the USA in performing economic evaluation studies in HIV/AIDS. Moreover, we applied an advanced method to understand thoroughly the content of keywords and abstracts, indicating the development of research domains as well as landscapes in this field and exploring the research gaps. The findings would be used to recommend further directions in order to advance this research area.

By analyzing the contents of abstracts, we could identify the most common economic evaluation research topics in the field of HIV/AIDS. Economic evaluation had been applied in various topics, including prevention (such as harm reduction, condom use or pre and post-exposure prophylaxis); HIV-related clinical services (testing and counselling services; ART, or vaccine); or co-infections (tuberculosis, hepatitis C, or HPV). Nonetheless, these results also imply some research gaps that have not been fully investigated. For instance, little economic evaluation evidence was found for transgender or migrant people, who are at very high risk of HIV infection [60, 61]. Also, there was a lack of economic evaluation evidence in structural interventions such as policy experiments or integrated/decentralized HIV-related service models, especially for stigmatized populations such as men who have sex with men [8, 62]. As infecting HIV requires lifelong treatment, evaluations in effectiveness and cost-effectiveness of interventions reducing the burden of aging and non-communicable diseases in HIV patients are also necessary [63–65]. Furthermore, our results showed a lack of economic evaluation evidence on the intervention using theory-driven eHealth approach, which is increasingly performed to prevent HIV risk behaviors, promote HIV testing and support HIV treatment [66, 67]. Last, but not least, the studies on the affordability were lacking. Policymakers with short-term constrained resources may not be able to invest in the high-cost interventions despite their superior health benefits [68]. Therefore, information about the affordability, feasibility and acceptability of the interventions should be incorporated into the economic evaluation studies. This information will support policymakers optimally allocating the available resource.

In this study, we found that CEA was the most common analytical method for economic evaluation, which is in line with other studies investigating the patterns of economic evaluations in general [69] and in pediatric research [70]. CEA uses disease-specific clinical, biomedical indicators or non-compound measures of survival as outcomes, for example, cost per infection averted/prevented or cost per life year saved; hence, contextualized features are not fully reflected [12]. Meanwhile, CUA is the gold standard for economic evaluation, since it uses utility-based units as outcomes (such as cost per QALYs gained or DALYs averted), which enables the comparison of various type of interventions and disease conditions. A previous review indicated that QALYs were more popular in high income countries, while low and low-middle income countries mostly used DALYs averted [71]. A key benefit of the utility-based units CUA uses is that they consider both the patients’ quality of life/health and the effects of interventions on mortality, which are more important than purely clinical outcomes often used in CEA. However, in practice, many studies in the field of HIV/AIDS reported both clinical outcomes and QALYs/DALYs outcomes; and they used the term “cost-effectiveness analysis” instead of “cost-utility analysis” [7, 8, 17, 24–26]. In CBA it is necessary to quantify the monetary value of the outcomes of healthcare interventions. This is complex and some consider it to be unethical to place a monetary value on human life. These disadvantages hinder the use of CBA and CEA/CUA studies have become the dominant method.

Our findings also revealed a discrepancy in the geographical distribution of economic evaluation studies. The current results illustrate a high fragmentation of economic evaluation studies, which were mostly performed by authors in a few countries such as the USA (concentrated epidemic), England (concentrated epidemic), Canada (concentrated epidemic) and South Africa (Hyperendemic). Among top 20 countries as study settings having the highest number of economic evaluation publications, only five countries had severe HIV epidemic including South Africa (hyperendemic), Uganda (generalized), Kenya (generalized), Tanzania (generalized), and Zambia (generalized). Indeed, the application of economic evaluation studies for HIV responses highly depends on socioeconomic, epidemiological and health system characteristics in each nation. Jacobsen et al. argued that most of the economic evaluation models examining HIV/AIDS interventions were heterogeneous due to variations of methodologies and settings [68]. Insufficient local cost and effectiveness data were one of the major reasons that obstructed the utilization of economic evaluation research in policy-making process, leading to incorrect decisions in selecting priorities [72]. This lost benefit might even be greater in LMICs than that in high-income countries [69]. The Second Panel on Cost-effectiveness in Health and Medicine in 2016 suggested that interventions’ impacts on both health and non-health outcomes (such as work productivity, education, or environment) should be measured to reflect fully the societal perspectives of interventions in specific settings [73]. Therefore, increasing the presence of contextualized data and strategies are vital to promoting the quality and applicability of economic evaluation studies in HIV/AIDS research.

The partnership network analysis in our study shows clearly that countries in similar geographical regions tended to collaborate in performing economic evaluation research. This result implies that building strong regional and sub-regional partnership networks between leading countries and other countries in similar regions or with similar contexts may be beneficial. For example, countries such as South Africa, India, China, Brazil and the United Kingdom could play a center role in bringing together available resources from other countries in the same regions to conduct multi-country economic evaluation studies. Such a regional initiative could substantially increase the quality of evidence as well as the research capacity of each country members in the network. Previous bibliometric analyses had agreements in the critically important role of international and local collaboration networks in enhancing research capacity and evidence transfer [29, 74].

Our study contains several limitations. First, our searching process restricted in using only the WoS albeit its advantages compared to other databases. The limited databases might restrict our ability to cover all possible health economic and economic evaluation studies in HIV/AIDS field. The misuse of keyword such as CEA instead of CUA could result in the underestimation in the volume of publications for each economic evaluation form. Second, we only included peer-reviewed publications in the English language; therefore, potential grey documents reporting health economic studies and economic evaluation were not considered. Furthermore, we could only analyze abstracts and keywords instead of full-text of publications, which might not capture the main themes of selected publications. Fourth, our analysis was not able to examine the transferability of each research, which referred to the application of economic evaluation evidence in one country to another country. Further research should evaluate the transferability and applicability of economic evaluation in the decision-making process in each country.

## Conclusion

In conclusion, this study highlighted the methodological and geographical discrepancies in health economic and economic evaluations in the field of HIV/AIDS. Developing regional collaboration networks and performing studies that incorporate affordability, feasibility and acceptability of the interventions would potentially improve the research capacity and study quality in economic evaluation research.

## Data Availability

The datasets supporting the conclusions of this article are included within the article and its additional file.

## Abbreviations

ART: Antiretroviral therapy
CBA: Cost-benefit analysis
CEA: Cost-effectiveness analysis
CUA: Cost-utility analysis
DALY: Disability-adjusted life years
LDA: Latent Dirichlet Allocation
LMICs: Low- and middle-income countries
PLWH: People living with HIV
QALY: Quality-adjusted life years
STIs: Sexually Transmitted Infections
UNAIDS: Joint United Nations Programme on HIV/AIDS
USA: United States of America
WoS: Web of Science
